# Lessons Learned: Implementation & Sustainment of a Geriatric Care Model Within a Changing Healthcare Landscape

**DOI:** 10.1093/geroni/igaf122.1230

**Published:** 2025-12-31

**Authors:** Michelle Rossi, Steve Barczi, William Hung

**Affiliations:** VA Pittsburgh Health Care System, Pittsburgh, Pennsylvania, United States; Veterans Health Administration, Madison, Wisconsin, United States; James J Peters VA Medical Center, New York, New York, United States

## Abstract

Americans in rural communities tend to be older and in poorer health than their urban counterparts. Telemedicine programs like GRECC Connect, within the VA healthcare system, have proven to reduce healthcare barriers such as limited availability of specialists in rural areas, cost, and travel time for frail older patients. During the initiation of this program, telehealth was widely supported within VA. Even with this support, implementation and dissemination was a challenge with rural staff turnover and limitations of technology and Wi-Fi access as examples. GRECC Connect has become a mature program which has been focusing in recent years on maintaining the positive gains GRECC Connect has made in educating rural primary care clinicians and caring for older medically complex rural Veterans despite the changing landscape of healthcare inside and outside of VA. We will discuss lessons learned in our efforts to sustain GRECC Connect clinical efforts and how these may inform implementation and sustainment of Geriatric models in context of a Value based payment system.

